# Defining the Personal Determinants of Health for Older Adults

**DOI:** 10.1093/geroni/igaa057.3392

**Published:** 2020-12-16

**Authors:** Stephanie MacLeod, Sandra Kraemer, Annette Fellows, Laurie Albright, Joann Ruiz, Michael McGinn, James Schaeffer, Charlotte Yeh

**Affiliations:** 1 Optum, Ann Arbor, Michigan, United States; 2 UnitedHealthcare, Minneapolis, Minnesota, United States; 3 AARP Services, Inc, Dedham, Massachusetts, United States

## Abstract

Social Determinants of Health (SDOH) are defined as the broad community-scale conditions and factors shaping daily life. Meanwhile, less is known about the personal, individual-level characteristics impacting health outcomes. These have not been defined as a construct, leaving a gap in overall understanding of the resources that shape healthy and successful aging. Our primary purpose is to propose and define a new construct encompassing critical personal resources to be known as the Personal Determinants of Health (PDOH), built on resilience as a key strength later in life and supported by factors that buffer challenges and declining health. A literature search was conducted to streamline the scope of this review, with key terms determined to identify relevant publications; common databases and resources were utilized. Search strategies failed to identify a standard definition for Personal Determinants of Health as a construct, nor does this term exist regarding applied initiatives with older adults. Thus, a clear opportunity exists to establish PDOH as a unique construct. Despite evidence that individual characteristics impact late-life health outcomes, these key personal resources have not been established as a separate construct. Thus, we propose to define PDOH with a foundation of resilience supported by selected personal resources. Establishing this new construct will be critical in designing initiatives to support older adults and improve their health outcomes.

